# Deficiency of CD147 Attenuated Non-alcoholic Steatohepatitis Progression in an NLRP3-Dependent Manner

**DOI:** 10.3389/fcell.2020.00784

**Published:** 2020-08-13

**Authors:** Tian Zhang, Hao Li, Ke Wang, Bing Xu, Zhi-Nan Chen, Huijie Bian

**Affiliations:** ^1^National Translational Science Center for Molecular Medicine, Department of Cell Biology, Fourth Military Medical University, Xi’an, China; ^2^State Key Laboratory of Cancer Biology, National Clinical Research Center for Digestive Diseases, Xijing Hospital of Digestive Diseases, Fourth Military Medical University, Xi’an, China

**Keywords:** CD147, NASH, inflammation, NLRP3, CypA

## Abstract

Cluster of differentiation 147 (CD147) is a transmembrane glycoprotein belonging to the immunoglobulin superfamily. CD147 overexpression has been reported to facilitate the development of hepatocellular carcinoma (HCC) and influence immunologic disorders. Although increased expression of CD147 was reported in non-alcoholic steatohepatitis (NASH), functions of CD147 in NASH have not been evaluated. Firstly, we confirmed that CD147 expression was increased in the liver tissues from methionine-choline-deficient (MCD) diet-induced NASH model mice and NASH patients. Mice with hepatocyte-specific CD147 deletion exhibited attenuated NASH phenotypes, including reduced steatosis, liver injury, hepatocyte apoptosis and inflammatory cytokines IL-1β/IL-18 secretion. Following the administration of the MCD diet, NLRP3 expression was increased gradually along with CD147 expression. Furthermore, CD147 deletion inhibited the NF-κB/NLRP3 signaling pathway in both MCD diet-induced mice and primary hepatocytes. Finally, CypA inhibitor TMN355 attenuated liver steatosis and injury and inhibited NF-κB/NLRP3 signaling pathway. Therefore, our results suggest that CD147 played a vital role in NASH pathogenesis by regulating the inflammatory response, and CypA/CD147 could be attractive therapeutic targets for NASH treatment.

## Introduction

Non-alcoholic fatty liver disease (NAFLD) is one of the most common chronic liver diseases worldwide, with an estimated global prevalence of 25–45% (Rinella, 2015). The hepatic pathology of NAFLD is defined as a metabolic syndrome component ranging from simple steatosis to non-alcoholic steatohepatitis (NASH). NASH is characterized by hepatic steatosis with inflammation, hepatocyte apoptosis and fibrosis and can progress to more severe stages, such as cirrhosis and hepatocellular carcinoma (HCC) (Younossi et al., 2019). A “two-hit” hypothesis has been proposed to explain the pathogenesis of NASH. Lipid accumulation in the liver acts as the primary factor that initiates and propagates multiple events, including hepatocyte injury, oxidative stress, endoplasmic reticulum stress, mitochondrial dysfunction, and regulation of inflammation (Friedman et al., 2018). These findings suggest that the proinflammatory response in the liver is closely associated with hepatocellular death and progression from NASH to fibrosis (Schuster et al., 2018). Despite the high prevalence of NASH, its contributing factors remain poorly understood, and no treatment has proven effective.

Inflammasomes are cytoplasmic multiprotein complexes composed of nod-like-receptor (NLR) and pyrin and HIN domain-containing protein (PYHIN) (Schroder and Tschopp, 2010). These complexes are sensors of endogenous or exogenous pathogen-associated molecular patterns (PAMPs) or damage-associated molecular patterns (DAMPs) that govern and initiate the cleavage of the effector proinflammatory cytokines IL-1β and IL-18 via caspase-1 activation (Rathinam and Fitzgerald, 2016). In the NLRP3 inflammasome, the NLR component is represented by NLRP3, which forms a complex with the adaptor molecule ASC and caspase-1 (Mangan et al., 2018). Activation of the inflammasome is a two-step process; the primary step is upregulation of NLRP3 expression via activation of the NF-κB signaling pathway, and the second step depends on inflammasome ligands (Swanson et al., 2019). The role of NLRP3 inflammasome has been extensively studied in macrophage, which is the main source of pro-inflammatory cytokines including IL-1β and IL-18 (Szabo and Csak, 2012). We noticed that other cell types, including hepatic stellate cells and hepatocytes, were also involved in regulation of NASH progression by NLRP3 signal pathway (Szabo and Petrasek, 2015). Recently, substantial evidence has shown that NLRP3 inflammasome activation in hepatocytes plays an important role in liver injury, inflammation and fibrosis (Wree et al., 2014; Han et al., 2018).

Cluster of differentiation 147 (CD147), also known as basigin, is highly expressed on the surface of carcinoma cells (Yan et al., 2005). CD147 is a heavily glycosylated type I transmembrane glycoprotein, and its overexpression is significantly associated with various malignant tumors and poor prognosis (Li et al., 2009). Numerous studies suggest that CD147 contributes to the hallmarks of HCC by participating in carcinogenesis (Lu et al., 2018), metabolic reprogramming (Huang et al., 2014) and epithelial-to-mesenchymal transition (EMT) (Wu et al., 2011). Moreover, CD147 plays an important role in aspects of inflammation regulation, including neutrophil adhesion (Kato et al., 2009), chemotaxis (Wang et al., 2011), and oxidative stress (Kim et al., 2012). The regulatory function of CD147 is induced by binding with its ligand, cyclophilin A (CypA), which is implicated in various proinflammatory signaling pathways (Dawar et al., 2017). In rheumatoid arthritis, the adhesive and invasive abilities of neutrophils are upregulated by the CypA/CD147 complex, and CypA inhibition reduces the number of inflammatory cells (Yang et al., 2008). These results suggest that the interaction of CypA and CD147 could be used as a potential biomarker for diverse inflammatory diseases.

Cluster of differentiation 147 expression was reported to be upregulated in NASH, while the function of CD147 and its mechanism in NASH have not been addressed (Thomas et al., 2013). Moreover, there is no evidence currently available on whether the CypA/CD147 complex may participate in proinflammatory signaling in liver disease and represent a new therapeutic intervention for NASH. In the present study, we demonstrated that CD147 is the driving factor of NASH pathogenesis in experimental mouse models and determined the role of CD147 in methionine-choline-deficient (MCD) diet-induced hepatocytes. In particular, we elucidated the pivotal proinflammatory function of CD147 in NASH pathogenesis through its ligand CypA in mediating the NF-κB/NLRP3 signaling pathway. More importantly, counteracting CypA with small molecule inhibitors effectively rescued mice from MCD diet-induced NASH. These results suggested that CypA and CD147 are pathogenic factors in liver tissue inflammation in NASH and could be therapeutic targets in NASH patients.

## Materials and Methods

### Animal Models

All animal protocols were approved by the Institutional Animal Care and Use Committee of the Fourth Military Medical University (FMMU). Mice were maintained under specific pathogen-free conditions in a temperature-controlled environment and on a 12/12-h light/dark cycle at the Laboratory Animal Research Center. Hepatocyte-specific Bsg/CD147 deletion mice (Alb;Bsg^flx/flx^ mice) were generated by Dr. Wu in our laboratory (Wu et al., 2016). To establish murine NASH model, 8-week-old male C57BL/6J mice were randomly fed a normal chow diet or an MCD diet for six weeks to establish NASH. In addition, 8-week-old male Alb;Bsg^flx/flx^ mice and male littermate control Bsg^flx/flx^ mice were fed the same diet for two weeks to induce NASH. For *in vivo* treatment with a CypA inhibitor, 8-week-old male mice were fed either an MCD diet or matched control diet for 2 weeks, and the MCD diet mice were randomly divided into four groups after 1 week. The mice were intraperitoneally administered with 10 mg/kg or 20 mg/kg TMN355 (Tocris, MN, United States, 4152) or dimethyl sulfoxide (DMSO) three times for 7 days.

### Human Subjects

Human liver tissue samples of normal, steatosis and NASH were collected from Xijing Hospital of FMMU for western blot analysis. Liver tissue biopsy samples were obtained from the Xijing Hospital of FMMU (five NAFLD patients and five healthy donors) and Alenabio Biotechnology (16 NAFLD patients). All patients provided written informed consent for analysis of their tissue for research purposes, and all experiments were performed with approval from the Clinical Research Ethics Committee of FMMU.

### Cell Line

MIHA cell line was obtained from Xijing Hospital of Digestive Diseases of FMMU (Xi’an, China), and cultured in Dulbecco’s Modified Eagle’s Medium (DMEM, Gibco, Grand Island, NY, United States, 11965092) supplemented with 10% fetal bovine serum (FBS, Gibco, 10100147). MIHA cells were transfected with pcDNA3.1 or pcDNA3.1-CD147 and treated with DMSO or ammonium pyrrolidinedithiocarbamate (PDTC, 100 μM, Abcam, Cambridge, United Kingdom, ab141406) for 24 h.

### Mouse Primary Hepatocyte Isolation and Culture

The method used for primary hepatocyte isolation was based on a two-step collagenase perfusion technique. In brief, hepatocytes were dissociated from anesthetized 8-week-old male mice by non-recirculating perfusion of collagenase IV (Sigma-Aldrich, St. Louis, MO, United States, C5138) through the portal vein. The isolated cells were then filtered through a nylon filter and centrifuged with Percoll (Solarbio, Beijing, China, P8370) solution. Finally, the viable cells at the bottom of the Percoll gradient were collected as primary hepatocytes and cultured in William’s E medium (Gibco, A1217601) or MCD medium (Caisson, Smithfield, UT, United States, WMP03-1LT).

### Histology and Staining Analysis

Paraffin-embedded liver tissue sections were routinely stained with hematoxylin and eosin (H&E) using standard protocols. Oil Red O staining was conducted on frozen liver tissue sections with a commercial kit (Nanjing Jiancheng Bioengineering, Nanjing, China, D027). A FragEL DNA Fragmentation Detection Kit (Merck Millipore, Billerica, MA, United States, QIA39) was used for TUNEL. For immunohistochemistry, liver sections were stained with primary antibodies against CD147 (Abcam, ab34016). All images were acquired using a Nikon microscope.

### Western Blotting

Western blotting was performed as previously described. Tissues and cultured cells were lysed in RIPA buffer (Beyotime Biotechnology, Shanghai, China, P0013B) supplemented with 1 mM PMSF (Beyotime Biotechnology, ST505) for 30 min on ice. Protein extracts were obtained by centrifugation for 30 min at 4°C. Proteins were separated on SDS-PAGE gels and transferred to PVDF membranes. Membranes were then incubated with antibodies against CD147 (R&D Systems, MN, United States, AF772), NLRP3 (R&D Systems, MAB7578), Bcl-2 (Huabio, Hangzhou, China, M1206-4), Bax (Huabio, ER0907), p65 (Proteintech, Wuhan, China, 10745-1-AP), p-p65 (Cell Signaling Technology, CA, United States, 3033S), Lamin B (Proteintech, 66095-1) or α-Tubulin (Proteintech, 66031-1) in TBST. After incubation of membranes with secondary antibodies, protein bands were visualized with ECL solution (Beyotime Biotechnology, Shanghai, China, P0018FS). Image analysis procedures were performed with Carestream Molecular Image software.

### Real-Time PCR

Total RNA was extracted from liver tissues or primary hepatocytes using a Total RNA Kit II (Omega, Norcross, GA, United States, R6934) according to the manufacturer’s instructions. The cDNA was synthesized using Prime Script RT Reagent Kit (Takara, Tokyo, Japan, DRR037A). Real-time PCR was conducted using SYBR Premix Ex Taq (Takara, DRR081A). The results were calculated using the 2−ΔΔCt method. The following primers were used in this study. CD147: Forward 5′-GGCTGGTTTCCTCAAGGCA-3′, Reverse 5′-TAG GCGGCATGGATGTGAAC-3′; NLRP3: Forward 5′-ATTACC CGCCCGAGAAAGG, Reverse 5′-TCGCAGCAAAGATCCACA CAG; 18S: Forward 5′-GTAACCCGTTGAACCCCATT-3′, Reverse 5′-CCATCCAATCGGTAGTAGCG-3′; IL-1β: Forward 5′-TGGGCCTCAAAGGAAAGAAT-3′, Reverse 5′-CTTGGGA TCCACACTCTCCA-3′; IL-18: Forward 5′-GACTCTTGCGTC AACTTCAAGG-3′, Reverse 5′-CAGGCTGTCTTTTGTCAAC GA-3′; MCP-1: Forward 5′-AGCAGCAGGTGTCCCAAAGA-3′, Reverse 5′-GTGCTGAAGACCTTAGGGCAGA-3′; TNF-α: Forward 5′-CGTGCTCCTCACCCACAC-3′, Reverse 5′-GGGT TCATACCAGGGTTTGA-3′; Caspase1: Forward 5′-ACAAGG CACGGGACCTATG-3′, Reverse 5′-TCCCAGTCAGTCCTGGA AATG-3′.

### Biochemical Analysis and Cytokine Measurement

The serum alanine aminotransferase (ALT, C009-2-1), aspartate aminotransferase (AST, C010-2-1) levels and liver triglyceride (TG, A110-1-1) content were determined using commercial kits from Nanjing Jiancheng Bioengineering. Serum IL-1β level was measured with ELISA kits (Dakewe, Shenzhen, China, DKW12-2012). Serum CypA (orb365382) and IL-18 (orb366030) levels were measured with ELISA kits from Biorbyt (Cambridge, United Kingdom).

### Isolation of Nuclear and Cytoplasmic Extracts

The nuclear extract was prepared using an NE-PER Nuclear Cytoplasmic Extraction Reagent kit (Thermo, Waltham, MA, United States, 78835) according to the manufacturer’s instructions.

### Statistical Analysis

All data were expressed as the mean ± SEM and were analyzed using either one-way analysis of variance or two-tailed unpaired Student’s *t*-test. For each data parameter presented, ^∗^ indicated *p* < 0.05 and ^∗∗^ indicated *p* < 0.01. All analyses were performed using GraphPad Prism Version 5 software.

## Results

### CD147 Expression Was Enhanced in the Liver of MCD Diet-Induced Mice

**FIGURE 1:**
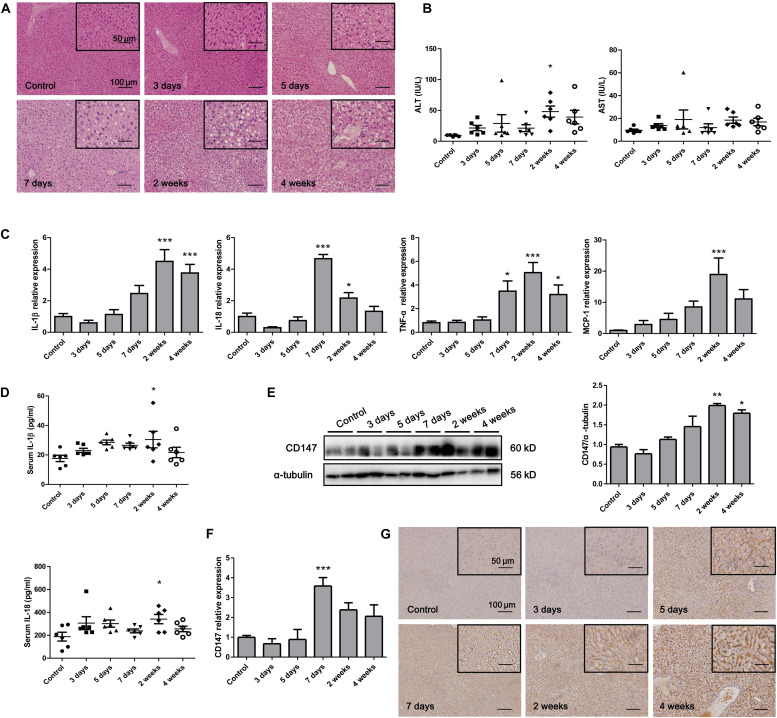
CD147 was increased during NASH progression in MCD diet-induced C57/BL6 mice. **(A)** Representative H&E staining images of tissues from NASH model mice. **(B)** Serum ALT and AST levels in NASH model mice (*n* = 6). **(C)** The mRNA levels of inflammatory cytokines in liver tissues from mice with NASH (*n* = 6). **(D)** Serum IL-1β and IL-18 levels in NASH model mice (*n* = 6). **(E)** Protein levels of CD147 in liver tissues from mice with NASH (*n* = 2). **(F)** The mRNA levels of CD147 in liver tissues from mice with NASH (*n* = 6). **(G)** IHC staining for CD147 in liver tissues of NASH model mice. One-way ANOVA followed by Dunnett’s test was used to compare NASH model mice with normal group. *p < 0.05, **p < 0.01, and ***p < 0.001.

To analyze CD147 expression during the progression of NASH in an animal model, we applied the MCD diet-induced NASH mouse model. H&E staining indicated that the accumulation of hepatic lipids was increased in the MCD diet group compared with the control group (Figure 1A). The liver injury parameters, serum ALT and AST were increased in the MCD diet group, in which the level of the damage-sensitive factor ALT was significantly increased at 2 weeks (Figure 1B). Moreover, the mRNA levels of inflammatory cytokines, including IL-1β, IL-18, TNF-α, and MCP-1, were increased in the liver tissues of the MCD diet group (Figure 1C). Accordingly, serum IL-1β and IL-18 were significantly upregulated at 2 weeks (Figure 1D). Interestingly, increased CD147 expression was observed in liver tissues of the MCD diet group, as indicated by western blotting and qPCR (Figures 1E,F). IHC analysis also showed increased CD147 expression in the liver by MCD diet induction (Figure 1G).

### CD147 Expression Was Enhanced in Liver Tissues From NAFLD Patients

**FIGURE 2:**
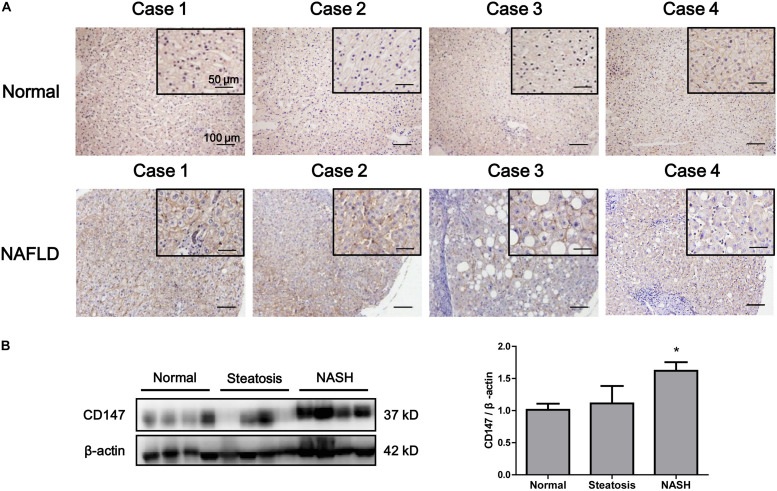
Increased expression of CD147 in liver tissues from NAFLD patients. **(A)** IHC staining for CD147 in human liver tissues from NAFLD patients and healthy donors. **(B)** Protein levels of CD147 in liver tissues from normal, steatosis, and NASH patients. Band relative intensity was normalized by β-actin, and unpaired *t* test was used for statistical analysis. *p < 0.05.

We evaluated the expression of CD147 in liver biopsies from NASH patients, steatosis patients and control subjects. CD147 expression was significantly increased in NAFLD samples, as determined by immunohistochemistry (Figure 2A), with a positive rate of 76.2% (Table 1). Moreover, western blot analysis indicated CD147 expression was upregulated significantly in NASH tissues and slightly in steatosis tissues (Figure 2B).

**TABLE 1:** CD147 expression in liver steatosis tissues from patients.

Type of liver tissue	Positive/total cases	Positive rate	*p* value
Normal	1/5	20%	0.034
NAFLD	16/21	76.2%	

### Hepatocyte-Specific Deletion of CD147 Attenuated NASH Progression

**FIGURE 3:**
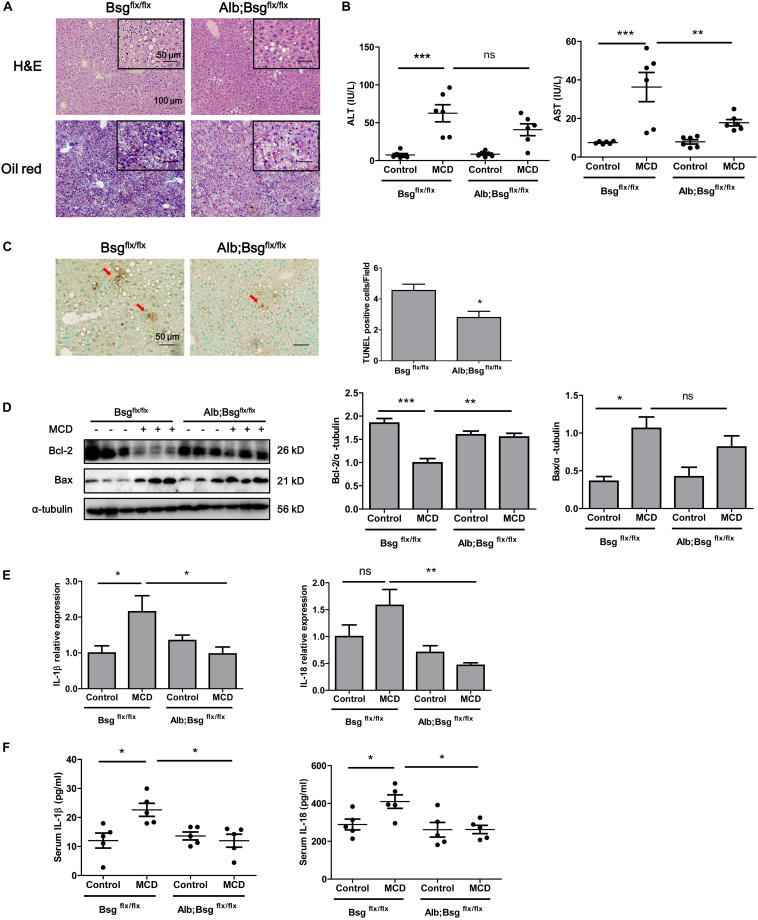
CD147 hepatocyte-specific deletion impeded NASH progression in MCD diet-fed mice. Bsg^flx/flx^ and Alb;Bsg^flx/flx^ mice were fed an MCD or control diet for 2 weeks. **(A)** Representative images of H&E and Oil Red O staining of liver sections. **(B)** Serum ALT and AST levels (*n* = 6). One-way ANOVA followed by Bonferroni’s multiple comparison test was used to compare indicated group. **p < 0.01, ***p < 0.001. **(C)** Representative TUNEL images of liver sections and numbers of apoptotic TUNEL-positive cells (*n* = 6). Unpaired *t* test was used to compare apoptotic cell numbers. *p < 0.05. **(D)** Hepatic protein levels of Bax and Bcl-2. Band relative intensity was normalized by α-tubulin, and One-way ANOVA followed by Bonferroni’s multiple comparison test was used for statistical analysis. *p < 0.05, **p < 0.01, and ***p < 0.001. **(E)** mRNA levels of IL-1β and IL-18 (*n* = 5). One-way ANOVA followed by Bonferroni’s multiple comparison test was used to compare each group. *p < 0.05, **p < 0.01. **(F)** Serum levels of IL-1β and IL-18 (*n* = 5). One-way ANOVA followed by Bonferroni’s multiple comparison test was used to compare each group. *p < 0.05.

To clarify the involvement of CD147 in NASH progression, we generated Alb;Bsg^flx/flx^ mice and fed with MCD diet for 2 weeks. H&E and Oil Red O staining indicated a decreased lipid accumulation in liver tissues of Alb;Bsg^flx/flx^ mice compared with those of littermate mice (Figure 3A). Serum ALT and AST results showed that liver injury was significantly induced by MCD diet feeding, but this effect was reversed in Alb;Bsg^flx/flx^ mice, indicating that liver injury was attenuated by CD147 knockout (Figure 3B). TUNEL assays showed a decreased number of apoptotic cells in liver tissues of MCD diet-induced Alb;Bsg^flx/flx^ mice compared with MCD diet-induced Bsg^flx/flx^ mice (Figure 3C). Consistent with this result, the level of the anti-apoptotic protein Bcl-2 was decreased in MCD diet-induced littermate mice but was restored in Alb;Bsg^flx/flx^ mice; while the increased expression of proapoptotic protein Bax in the littermate control group was reversed in Alb;Bsg^flx/flx^ mice (Figure 3D). In addition, the elevation in the levels of the inflammatory cytokines IL-1β and IL-18 induced by MCD diet feeding was significantly reduced in liver tissues and serum samples of Alb;Bsg^flx/flx^ mice (Figures 3E,F). Taken together, these data suggested that hepatocyte-specific deletion of CD147 impeded the progression of NASH by disrupting the inflammatory response.

### CD147 Deletion Inhibited the NLRP3 Signaling Pathway in MCD Diet-Induced Mice

**FIGURE 4:**
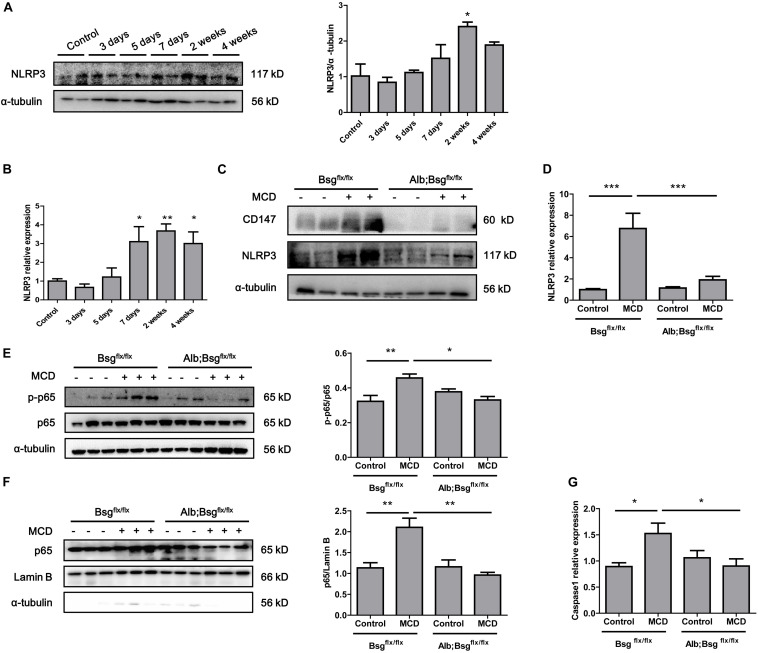
CD147 deficiency in hepatocytes inhibited activation of the NF-κB/NLRP3 signaling pathway. **(A)** Protein levels of NLRP3 in liver tissues of C57/BL6 NASH model mice (*n* = 2). Band relative intensity was normalized by α-tubulin, and one-way ANOVA followed by Dunnett’s test was used for statistical analysis. *p < 0.05. **(B)** mRNA levels of NLRP3 in liver tissues of C57/BL6 NASH model mice (*n* = 6). One-way ANOVA followed by Dunnett’s test was used for statistical analysis. *p < 0.05 and **p < 0.01. **(C)** Protein levels of NLRP3 in Bsg^flx/flx^ and Alb;Bsg^flx/flx^ mice fed control or MCD diet (*n* = 2). **(D)** mRNA levels of NLRP3 in Bsg^flx/flx^ and Alb;Bsg^flx/flx^ mice fed control or MCD diet (*n* = 5). One-way ANOVA followed by Bonferroni’s multiple comparison test was used to compare each group. ***p < 0.001. **(E)** Protein levels of p65 and p-p65 in Bsg^flx/flx^ and Alb;Bsg^flx/flx^ mice fed control or MCD diet (*n* = 3). One-way ANOVA followed by Bonferroni’s multiple comparison test was used to compare each group. *p < 0.05 and **p < 0.01. **(F)** Protein levels of nuclear p65 in Bsg^flx/flx^ and Alb;Bsg^flx/flx^ mice fed control or MCD diet (*n* = 3). One-way ANOVA followed by Bonferroni’s multiple comparison test was used to compare each group. **p < 0.01. **(G)** mRNA levels of Caspase1 in Bsg^flx/flx^ and Alb;Bsg^flx/flx^ mice fed control or MCD diet (*n* = 6). One-way ANOVA followed by Bonferroni’s multiple comparison test was used to compare each group. *p < 0.05.

Secretion of the proinflammatory cytokines IL-1β and IL-18 is regulated by NLRP3 inflammasome activation. To investigate whether hepatocyte-specific deletion of CD147 reduced the secretion of proinflammatory cytokines through the regulation of NLRP3 expression, we first detected the expression of NLRP3 in the MCD diet-induced mouse model. Western blotting and real-time PCR revealed increased expression of NLRP3 in liver tissues as NASH progressed (Figures 4A,B). Then, we used mice with hepatocyte-specific CD147 deletion to determine whether CD147 deficiency could inhibit NLRP3 inflammasome activation. Compared with MCD diet-induced littermate mice, Alb;Bsg^flx/flx^ mice fed the MCD diet showed decreased NLRP3 protein expression (Figure 4C). Similarly, mRNA levels of NLRP3 were significantly reduced in MCD diet-induced Alb;Bsg^flx/flx^ mice (Figure 4D). As the NF-κB signaling pathway plays a pivotal role in the pathogenesis of steatohepatitis and the activation of the NLRP3 inflammasome, the effect of CD147 on NF-κB activation was investigated. Phosphorylation of p65 (p-p65) was significantly increased in MCD diet-induced littermate mice, while the increased expression of p-p65 was reversed in MCD diet-induced Alb;Bsg^flx/flx^ mice (Figure 4E). Nuclear and cytoplasmic proteins were separately extracted from liver tissues. In the MCD diet-induced group, the increased p65 expression in the nucleus was reduced in Alb;Bsg^flx/flx^ mice compared with littermate mice (Figure 4F). In addition, the caspase1 mRNA level was reduced in the liver tissue of Alb;Bsg^flx/flx^ mice (Figure 4G). These data suggested that CD147 is involved in NLRP3 activation through an NF-κB-dependent pathway in MCD diet-induced NASH.

### CypA Regulated the NF-κB/NLRP3 Signaling Pathway via CD147 in Hepatocytes

**FIGURE 5:**
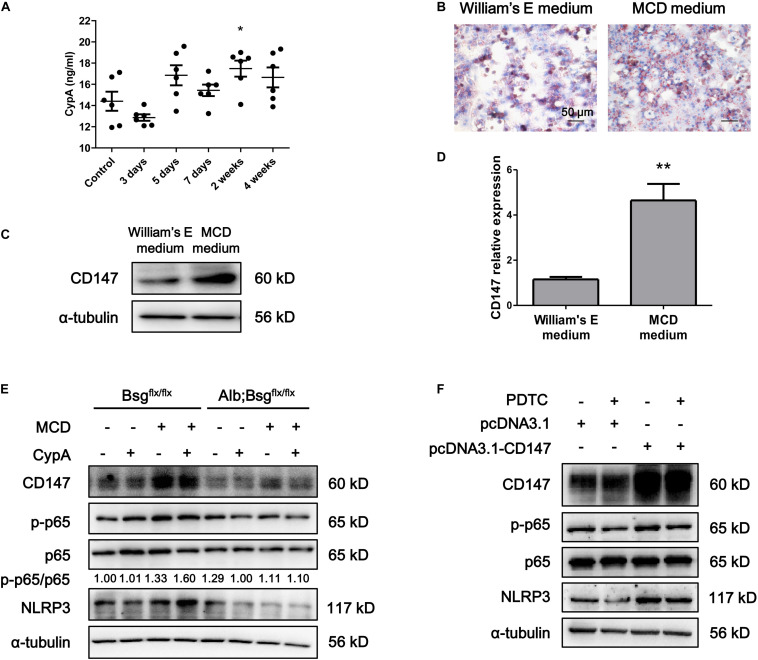
CD147 deficiency in hepatocytes regulated NLRP3 inflammasome activation due to the induction of CypA. **(A)** Serum levels of CypA in C57/BL6 NASH model mice (*n* = 6). One-way ANOVA followed by Dunnett’s test was used for statistical analysis. *p < 0.05. **(B)** Representative images of Oil Red O staining of primary hepatocytes isolated from C57/BL6 mice. **(C)** Protein levels of CD147 in primary hepatocytes isolated from C57/BL6 mice. **(D)** mRNA levels of CD147 in primary hepatocytes isolated from C57/BL6 mice. Unpaired *t* test was used for statistical analysis. **p < 0.01. **(E)** Primary hepatocytes were isolated from Bsg^flx/flx^ or Alb;Bsg^flx/flx^ mice and treated with CypA (2 μg/ml) and/or MCD medium for 24 h. Protein levels of p-p65, p65, NLRP3, and CD147 were detected. **(F)** MIHA cells were transfected with pcDNA3.1 or pcDNA3.1-CD147 and treated with DMSO or PDTC (100 μM) for 24 h. Protein levels of p-p65, p65, NLRP3, and CD147 were detected.

Cluster of differentiation 147 is the receptor for CypA, and the CypA/CD147 complex acts as a pivotal proinflammatory signaling pathway mediator. We first evaluated the serum expression of CypA in MCD diet-fed mice and found an increased tendency as NASH progressed (Figure 5A). Then, mouse primary hepatocytes were isolated and cultured in William’s E medium or MCD medium. Oil Red O staining indicated that lipid drops markedly accumulated in MCD medium-cultured primary hepatocytes (Figure 5B). Western blotting and real-time PCR showed that CD147 expression was increased in MCD medium-cultured primary hepatocytes compared with control group (Figures 5C,D). After CypA was added to MCD medium, the NLRP3 and p-p65/p65 protein levels were increased in primary hepatocytes of Bsg^flx/flx^ mice but remained unchanged in hepatocytes of Alb;Bsg^flx/flx^ mice (Figure 5E). The expression of p-p65 and NLRP3 was increased after CD147 overexpression, while the increased NLRP3 expression was reversed by NF-κB inhibitor PDTC, indicating that CD147 regulated NLRP3 expression in a NF-κB-dependent manner (Figure 5F). These results demonstrated that CypA and CD147 regulate the NF-κB/NLRP3 signaling pathway in MCD diet-induced NASH.

### CypA Inhibitor Alleviated MCD Diet-Induced NASH

**FIGURE 6:**
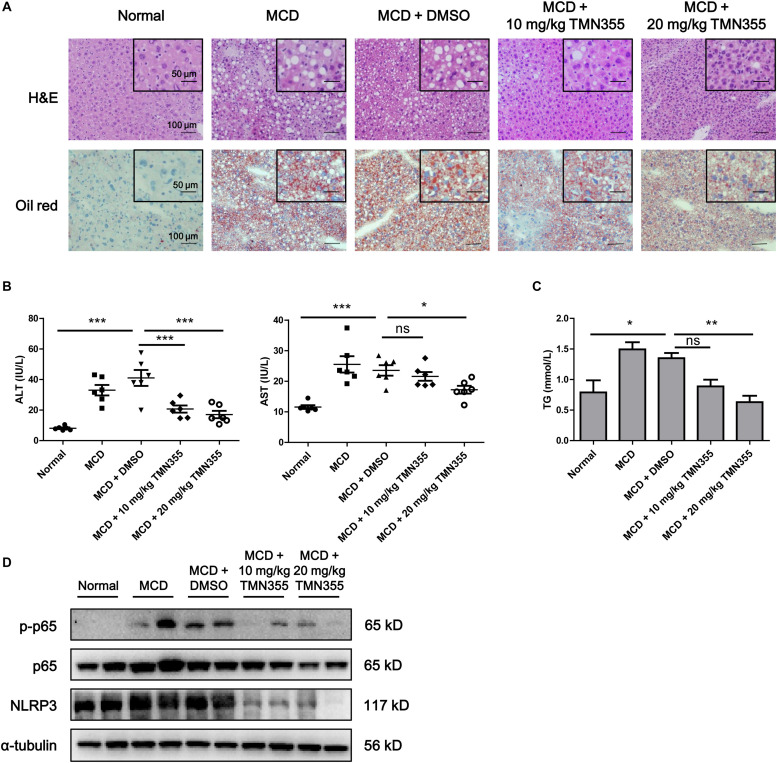
The CypA inhibitor TMN355 attenuated the severity of MCD diet-induced NASH in mice. Starting 7 days after MCD feeding, C57/BL6 mice were treated with TMN355 (10 and 20 mg/kg) or vehicle (DMSO) three times weekly before sacrifice. **(A)** Representative images of H&E and Oil Red O staining of liver sections. **(B)** Serum ALT and AST levels (*n* = 6). **(C)** Liver TG content (*n* = 6). One-way ANOVA followed by Dunnett’s test was used to compare each group with MCD + DMSO group. *p < 0.05, **p < 0.01, and ***p < 0.001. **(D)** Protein levels of NLRP3, p65 and p-p65 in MCD diet-induced C57/BL6 mice that were treated with TMN355 or DMSO.

Finally, to investigate the therapeutic role of CypA, MCD diet-fed mice were intraperitoneally administered with a CypA inhibitor, TMN355. As indicated by H&E and Oil Red O staining, hepatic lipid accumulation was increased in the MCD diet group, but this accumulation was alleviated after TMN355 treatment (Figure 6A). Similarly, the liver injury parameters ALT and AST were increased in the MCD diet group but decreased after TMN355 treatment (Figure 6B). Additionally, the increase in TG content in the liver tissues of the MCD diet group was blocked after TMN355 treatment (Figure 6C). Expression of NLRP3 and p-p65 was significantly increased in MCD diet-induced mice, while the increased expression of NLRP3 and p-p65 was suppressed by TMN355 treatment (Figure 6D). Therefore, these data suggested that the CypA inhibitor ameliorated MCD diet-induced NASH by inhibiting NF-κB/NLRP3 signaling pathway.

## Discussion

Inflammation is a common trigger of liver disease and is considered the main driver of liver tissue damage leading to fibrosis and HCC (Ringelhan et al., 2018). Much evidence has been presented to indicate the contribution of inflammation activation under non-alcoholic liver disease conditions (Ibrahim et al., 2018). CD147 is a widely expressed membrane glycoprotein that plays an important role in the inflammatory response and regulates the activity of the downstream NF-κB signaling pathway (Jin et al., 2017). Although NASH has been reported to be frequently associated with inflammation, to our knowledge, the functional importance of CD147 in NASH and its related mechanism have not been evaluated. Previous study has reported that CD147 expression was increased in the lard/cholesterol/sodium cholate diet-induced NASH mice (Thomas et al., 2013). Similarly, our results indicated the increased expression of CD147 in the MCD diet-induced NASH mice. Moreover, increased expression of CD147 was also observed in the liver tissues from NAFLD patients. Importantly, mice with hepatocyte-specific CD147 deletion fed an MCD diet exhibited significantly attenuated NASH phenotypes compared with control mice fed the same diet. These results suggest that CD147 played a crucial role in aggravating NASH progression in mice.

The accumulation of intracellular lipids leads to lipotoxicity, which is a characteristic predisposing factor for NAFLD and NASH (Marra and Svegliati-Baroni, 2018). Increased accumulation of harmful lipids in hepatocytes eventually leads to cell injury, death and the activation of inflammatory pathways (Ertunc and Hotamisligil, 2016). Generally, HFD model and MCD induced mouse model are two common models in NASH studies that indicate complementary characteristics. HFD model is a very reliable model to induce simple hepatic steatosis accompanied by metabolic syndrome including obesity, glucose intolerance and insulin resistance; however, liver damage, inflammation and fibrosis are seldom observed in most mouse strains (Carlessi et al., 2019). Although MCD induced mouse model lacks of physiological hallmarks of the metabolic syndrome which is associated with an increased risk for human NASH, it is adapted to study mechanisms of NASH-related liver injury, inflammation and progressive fibrosis (Wree et al., 2014). Based on characteristics of two mouse models, the present study using the MCD diet induced model mainly focused on the role of CD147 and its ligands in liver injury, inflammation and steatosis instead of metabolic syndrome.

Cluster of differentiation 147 was reported to play important roles in both parenchymal and non-parenchymal cells. The molecule mediated the activation of hepatic stellate cells by TGF-β1-CD147 positive feedback loop to promote liver fibrosis (Zhang et al., 2012; Li H. Y. et al., 2015). CD147 also promoted the phenotype differentiation, reactive oxygen species generation, and migration in macrophages (Winchester et al., 2015; Bauernfeind et al., 2009; Teymournejad and Rikihisa, 2020). In parenchymal cells, CD147 activated the Akt/mTOR signaling pathway and subsequently upregulated SREBP1c, leading to an increase in the transcription of major lipogenic genes to promote lipogenesis in HCC cells (Li J. et al., 2015). In the present study, we showed that CD147 deficiency in hepatocytes led to reduced hepatic lipid accumulation. Conceivably, CD147-promoted NASH may be related to the regulation of hepatic lipogenesis, although much evidence is needed to investigate this possibility. Cell death seems important in the progression of NASH, and several inhibitors of apoptosis have been suggested as potential treatments for NASH (Schwabe and Luedde, 2018). The previous study indicated that TUNEL-positive hepatocytes were significantly increased in the livers of NASH patients (Feldstein et al., 2003). It was reported that NASH patients had significantly decreased levels of the antiapoptotic protein Bcl-2 and that the degree of apoptosis was inversely correlated with the Bcl-2 level (El Bassat et al., 2014). Our TUNEL results revealed that CD147 knockout in hepatocytes reduced the number of apoptotic hepatic cells and decreased the Bcl-2 protein level, indicating that CD147 may participate in the apoptosis signaling pathway in NASH.

A dysregulated cytokine balance after liver injury can result in the aggravation of NASH development (Carter-Kent et al., 2008). We found that IL-1β and IL-18 were significantly reduced in Alb;Bsg^flx/flx^ mice fed the MCD diet compared with Bsg^flx/flx^ mice, suggesting that CD147 influences the inflammatory response by regulating IL-1β and IL-18 expression. In liver disease, IL-1β promotes the recruitment of inflammatory cells to the liver and induces TG accumulation in hepatocytes (Miura et al., 2010). Unlike the observations for IL-1β signaling, an increase in NASH severity was observed in IL-18-deficient mice with MCD diet-induced NASH. IL-18 deficiency altered the gut bacterial composition and resulted in inflammation (Henao-Mejia et al., 2012). The NLRP3 inflammasome is considered a platform for activating caspase-1 and inducing the maturation of proinflammatory cytokines, including IL-1β and IL-18 (Franchi et al., 2009). Emerging evidence indicates that NLRP3 inflammasome activation in hepatocytes plays an important role in liver injury, inflammation, and fibrosis (Wree et al., 2014; Han et al., 2018). Therefore, the expression of hepatic NLRP3 during NASH development was investigated in the present study. We confirmed that hepatic NLRP3 expression was significantly increased in mice with MCD diet-induced NASH and reduced in Alb;Bsg^flx/flx^ mice fed the MCD diet compared with Bsg^flx/flx^ mice. Conceivably, CD147 participates in the NLRP3 signaling pathway in the NASH models. NF-κB regulates the expression of proinflammatory mediators and functions as a master regulator of several inflammatory pathways (Oeckinghaus et al., 2011). Several studies have shown that the NLRP3 signaling pathway is regulated by the activation of NF-κB (Bauernfeind et al., 2009).

**FIGURE 7:**
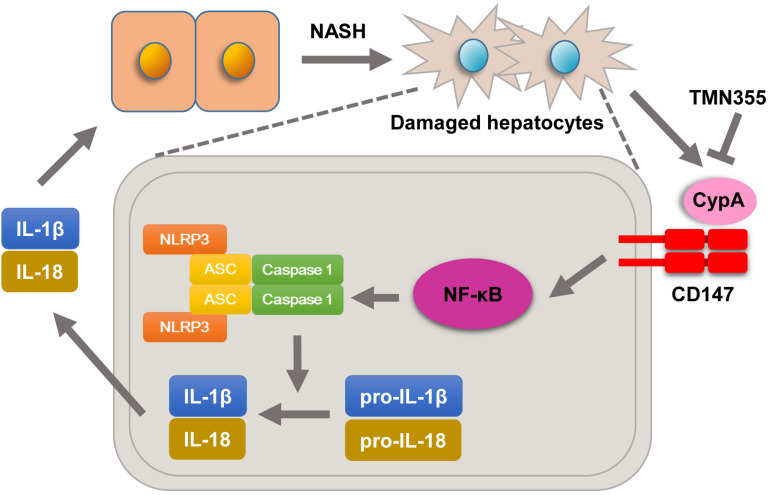
Molecular mechanisms by which CypA and CD147 contribute to NASH progression. Under MCD diet feeding, damaged hepatocytes release CypA, and CD147 expression is enhanced. The formation of the CypA/CD147 complex results in an inflammatory response through the NF-κB/NLRP3 signaling pathway.

Previous studies confirmed that the expression of CD147, as a surface receptor, is mediated, in part, through binding with extracellular CypA (Hoffmann and Schiene-Fischer, 2014). CypA was reported to be secreted into the extracellular environment in various cell types due to exposure to inflammatory stimuli (Dawar et al., 2017). Secreted CypA binds to CD147 and then results in NF-κB activation, chemotaxis, adhesion and migration (Qu et al., 2014). In this study, we showed that serum CypA expression was increased during the development of NASH. Importantly, we found that deleting CD147 from hepatocytes led to impaired NF-κB signaling in NASH mouse models. Furthermore, primary hepatocytes of Bsg^flx/flx^ mice treated with the CypA inhibitor exhibited increased expression of NLRP3 and NF-κB signaling activation when cultured in MCD medium. However, mice with hepatocyte-specific CD147 deletion did not exhibit this change. In seeking a possible mechanism underlying the effects of CypA on NASH, we found that treatment with the CypA inhibitor directly suppressed hepatic lipid accumulation in the MCD diet group. In addition, the liver injury parameters and TG content were decreased. These data suggested that NLRP3 activation promoted by CD147 was at least partly related to the regulation of CypA and NF-κB signaling (Figure 7). Notably, our study indicated that CypA could be a promising target for the treatment of NASH.

In conclusion, our work demonstrated that CD147 plays a key role in NASH pathogenesis via an NLRP3-dependent mechanism. CD147 deletion could be a potential treatment for NASH by mitigating hepatic steatosis, cell death and inflammation. Moreover, our findings provide new insight into the effects of CypA inhibitors on NASH and suggest an innovative therapeutic strategy.

## Data Availability Statement

The raw data supporting the conclusions of this article will be made available by the authors, without undue reservation.

## Ethics Statement

The studies involving human participants were reviewed and approved by Clinical Research Ethics Committee of FMMU. The patients/participants provided their written informed consent to participate in this study. The animal study was reviewed and approved by Institutional Animal Care and Use Committee of the Fourth Military Medical University.

## Author Contributions

TZ, Z-NC, and HB designed the study. TZ and HL performed the experiments. TZ, HL, KW, and BX acquired and analyzed the data. TZ drafted the original manuscript. TZ, HL, Z-NC, and HB revised the manuscript. All authors approved the final manuscript.

## Conflict of Interest

The authors declare that the research was conducted in the absence of any commercial or financial relationships that could be construed as a potential conflict of interest.

## References

[B1] Alexaki V. I. May A. E. Fujii C. Ungern-Sternberg S. N. Mund C. Gawaz M. (2017). S100A9 induces monocyte/macrophage migration via EMMPRIN. *Thromb. Haemost.* 117 636–639. 10.1160/th16-06-0434 27808344

[B2] Bauernfeind F. G. Horvath G. Stutz A. Alnemri E. S. MacDonald K. Speert D. (2009). Cutting edge: NF-kappaB activating pattern recognition and cytokine receptors license NLRP3 inflammasome activation by regulating NLRP3 expression. *J. Immunol.* 183 787–791. 10.4049/jimmunol.0901363 19570822 PMC2824855

[B3] Carlessi R. Kohn-Gaone J. Olynyk J. K. Tirnitz-Parker J. E. E. (2019). “Mouse models of hepatocellular carcinoma,” in *Hepatocellular Carcinoma*, ed. Tirnitz-Parker J. E. E. (Brisbane AU: Codon Publications Copyright: The Authors). 31664804

[B4] Carter-Kent C. Zein N. N. Feldstein A. E. (2008). Cytokines in the pathogenesis of fatty liver and disease progression to steatohepatitis: implications for treatment. *Am. J. Gastroenterol.* 103 1036–1042. 10.1111/j.1572-0241.2007.01709.x 18177455

[B5] Dawar F. U. Xiong Y. Khattak M. N. K. Li J. Lin L. Mei J. (2017). Potential role of cyclophilin A in regulating cytokine secretion. *J. Leukoc. Biol.* 102 989–992. 10.1189/jlb.3RU0317-090RR 28729360

[B6] El Bassat H. Ziada D. H. Hasby E. A. Nagy H. Abo Ryia M. H. (2014). Apoptotic and anti-apoptotic seromarkers for assessment of disease severity of non-alcoholic steatohepatitis. *Arab. J. Gastroenterol.* 15 6–11. 10.1016/j.ajg.2014.01.009 24630506

[B7] Ertunc M. E. Hotamisligil G. S. (2016). Lipid signaling and lipotoxicity in metaflammation: indications for metabolic disease pathogenesis and treatment. *J. Lipid Res.* 57 2099–2114. 10.1194/jlr.R066514 27330055 PMC5321214

[B8] Feldstein A. E. Canbay A. Angulo P. Taniai M. Burgart L. J. Lindor K. D. (2003). Hepatocyte apoptosis and fas expression are prominent features of human nonalcoholic steatohepatitis. *Gastroenterology* 125 437–443. 10.1016/s0016-5085(03)00907-7 12891546

[B9] Franchi L. Eigenbrod T. Munoz-Planillo R. Nunez G. (2009). The inflammasome: a caspase-1-activation platform that regulates immune responses and disease pathogenesis. *Nat. Immunol.* 10 241–247. 10.1038/ni.1703 19221555 PMC2820724

[B10] Friedman S. L. Neuschwander-Tetri B. A. Rinella M. Sanyal A. J. (2018). Mechanisms of NAFLD development and therapeutic strategies. *Nat. Med.* 24 908–922. 10.1038/s41591-018-0104-9 29967350 PMC6553468

[B11] Han C. Y. Rho H. S. Kim A. Kim T. H. Jang K. Jun D. W. (2018). FXR inhibits endoplasmic reticulum stress-induced nlrp3 inflammasome in hepatocytes and ameliorates liver Injury. *Cell Rep.* 24 2985–2999. 10.1016/j.celrep.2018.07.068 30208322

[B12] Henao-Mejia J. Elinav E. Jin C. Hao L. Mehal W. Z. Strowig T. (2012). Inflammasome-mediated dysbiosis regulates progression of NAFLD and obesity. *Nature* 482 179–185. 10.1038/nature10809 22297845 PMC3276682

[B13] Hoffmann H. Schiene-Fischer C. (2014). Functional aspects of extracellular cyclophilins. *Biol. Chem.* 395 721–735. 10.1515/hsz-2014-0125 24713575

[B14] Huang Q. Li J. Xing J. Li W. Li H. Ke X. (2014). CD147 promotes reprogramming of glucose metabolism and cell proliferation in HCC cells by inhibiting the p53-dependent signaling pathway. *J. Hepatol.* 61 859–866. 10.1016/j.jhep.2014.04.035 24801417

[B15] Ibrahim S. H. Hirsova P. Gores G. J. (2018). Non-alcoholic steatohepatitis pathogenesis: sublethal hepatocyte injury as a driver of liver inflammation. *Gut* 67 963–972. 10.1136/gutjnl-2017-315691 29367207 PMC5889737

[B16] Jin R. Xiao A. Y. Chen R. Granger D. N. Li G. (2017). Inhibition of CD147 (Cluster of Differentiation 147) Ameliorates Acute Ischemic Stroke in Mice by Reducing Thromboinflammation. *Stroke* 48 3356–3365. 10.1161/strokeaha.117.018839 29114092 PMC5726599

[B17] Kato N. Yuzawa Y. Kosugi T. Hobo A. Sato W. Miwa Y. (2009). The E-selectin ligand basigin/CD147 is responsible for neutrophil recruitment in renal ischemia/reperfusion. *J. Am. Soc. Nephrol.* 20 1565–1576. 10.1681/asn.2008090957 19443639 PMC2709679

[B18] Kim K. Kim H. Jeong K. Jung M. H. Hahn B. S. Yoon K. S. (2012). Release of overexpressed CypB activates ERK signaling through CD147 binding for hepatoma cell resistance to oxidative stress. *Apoptosis* 17 784–796. 10.1007/s10495-012-0730-5 22555451

[B19] Li H. Y. Ju D. Zhang D. W. Li H. Kong L. M. Guo Y. (2015). Activation of TGF-β1-CD147 positive feedback loop in hepatic stellate cells promotes liver fibrosis. *Sci Rep.* 5:16552. 10.1038/srep16552 26559755 PMC4642271

[B20] Li J. Huang Q. Long X. Zhang J. Huang X. Aa J. (2015). CD147 reprograms fatty acid metabolism in hepatocellular carcinoma cells through Akt/mTOR/SREBP1c and P38/PPARalpha pathways. *J. Hepatol.* 63 1378–1389. 10.1016/j.jhep.2015.07.039 26282231

[B21] Li Y. Xu J. Chen L. Zhong W. D. Zhang Z. Mi L. (2009). HAb18G (CD147), a cancer-associated biomarker and its role in cancer detection. *Histopathology* 54 677–687. 10.1111/j.1365-2559.2009.03280.x 19438743

[B22] Lu M. Wu J. Hao Z. W. Shang Y. K. Xu J. Nan G. (2018). Basolateral CD147 induces hepatocyte polarity loss by E-cadherin ubiquitination and degradation in hepatocellular carcinoma progress. *Hepatology* 68 317–332. 10.1002/hep.29798 29356040 PMC6055794

[B23] Mangan M. S. J. Olhava E. J. Roush W. R. Seidel H. M. Glick G. D. Latz E. (2018). Targeting the NLRP3 inflammasome in inflammatory diseases. *Nat. Rev. Drug Discov.* 17 588–606. 10.1038/nrd.2018.97 30026524

[B24] Marra F. Svegliati-Baroni G. (2018). Lipotoxicity and the gut-liver axis in NASH pathogenesis. *J. Hepatol.* 68 280–295. 10.1016/j.jhep.2017.11.014 29154964

[B25] Miura K. Kodama Y. Inokuchi S. Schnabl B. Aoyama T. Ohnishi H. (2010). Toll-like receptor 9 promotes steatohepatitis by induction of interleukin-1beta in mice. *Gastroenterology* 139 323.e7–34.e7. 10.1053/j.gastro.2010.03.052 20347818 PMC4631262

[B26] Oeckinghaus A. Hayden M. S. Ghosh S. (2011). Crosstalk in NF-kappaB signaling pathways. *Nat. Immunol.* 12 695–708. 10.1038/ni.2065 21772278

[B27] Qu X. Wang C. Zhang J. Qie G. Zhou J. (2014). The roles of CD147 and/or cyclophilin A in kidney diseases. *Mediators Inflamm.* 2014:728673. 10.1155/2014/728673 25580061 PMC4281390

[B28] Rathinam V. A. Fitzgerald K. A. (2016). Inflammasome complexes: emerging mechanisms and effector functions. *Cell* 165 792–800. 10.1016/j.cell.2016.03.046 27153493 PMC5503689

[B29] Rinella M. E. (2015). Nonalcoholic fatty liver disease: a systematic review. *JAMA* 313 2263–2273. 10.1001/jama.2015.5370 26057287

[B30] Ringelhan M. Pfister D. O’Connor T. Pikarsky E. Heikenwalder M. (2018). The immunology of hepatocellular carcinoma. *Nat. Immunol.* 19 222–232. 10.1038/s41590-018-0044-z 29379119

[B31] Schroder K. Tschopp J. (2010). The inflammasomes. *Cell* 140 821–832. 10.1016/j.cell.2010.01.040 20303873

[B32] Schuster S. Cabrera D. Arrese M. Feldstein A. E. (2018). Triggering and resolution of inflammation in NASH. *Nat. Rev. Gastroenterol. Hepatol.* 15 349–364. 10.1038/s41575-018-0009-6 29740166

[B33] Schwabe R. F. Luedde T. (2018). Apoptosis and necroptosis in the liver: a matter of life and death. *Nat. Rev. Gastroenterol. Hepatol.* 15 738–752. 10.1038/s41575-018-0065-y 30250076 PMC6490680

[B34] Swanson K. V. Deng M. Ting J. P. (2019). The NLRP3 inflammasome: molecular activation and regulation to therapeutics. *Nat. Rev. Immunol.* 19 477–489. 10.1038/s41577-019-0165-0 31036962 PMC7807242

[B35] Szabo G. Csak T. (2012). Inflammasomes in liver diseases. *J. Hepatol.* 57 642–654. 10.1016/j.jhep.2012.03.035 22634126

[B36] Szabo G. Petrasek J. (2015). Inflammasome activation and function in liver disease. *Nat. Rev. Gastroenterol. Hepatol.* 12 387–400. 10.1038/nrgastro.2015.94 26055245

[B37] Teymournejad O. Rikihisa Y. (2020). Ehrlichia chaffeensis uses an invasin to suppress reactive oxygen species generation by macrophages via CD147-dependent inhibition of Vav1 to block rac1 activation. *mBio* 11:e00267-20. 10.1128/mBio.00267-20 32317318 PMC7175088

[B38] Thomas A. Klein M. S. Stevens A. P. Reinders Y. Hellerbrand C. Dettmer K. (2013). Changes in the hepatic mitochondrial and membrane proteome in mice fed a non-alcoholic steatohepatitis inducing diet. *J. Proteomics* 80 107–122. 10.1016/j.jprot.2012.12.027 23313215

[B39] Wang C. H. Dai J. Y. Wang L. Jia J. F. Zheng Z. H. Ding J. (2011). Expression of CD147 (EMMPRIN) on neutrophils in rheumatoid arthritis enhances chemotaxis, matrix metalloproteinase production and invasiveness of synoviocytes. *J. Cell Mol. Med.* 15 850–860. 10.1111/j.1582-4934.2010.01084.x 20455995 PMC3922672

[B40] Winchester L. J. Veeranki S. Givvimani S. Tyagi S. C. (2015). Homocysteine elicits an M1 phenotype in murine macrophages through an EMMPRIN-mediated pathway. *Can. J. Physiol. Pharmacol.* 93 577–584. 10.1139/cjpp-2014-0520 26118387

[B41] Wree A. Eguchi A. McGeough M. D. Pena C. A. Johnson C. D. Canbay A. (2014). NLRP3 inflammasome activation results in hepatocyte pyroptosis, liver inflammation, and fibrosis in mice. *Hepatology* 59 898–910. 10.1002/hep.26592 23813842 PMC4008151

[B42] Wu J. Lu M. Li Y. Shang Y. K. Wang S. J. Meng Y. (2016). Regulation of a TGF-beta1-CD147 self-sustaining network in the differentiation plasticity of hepatocellular carcinoma cells. *Oncogene* 35 5468–5479. 10.1038/onc.2016.89 27041581

[B43] Wu J. Ru N. Y. Zhang Y. Li Y. Wei D. Ren Z. (2011). HAb18G/CD147 promotes epithelial-mesenchymal transition through TGF-beta signaling and is transcriptionally regulated by Slug. *Oncogene* 30 4410–4427. 10.1038/onc.2011.149 21532623

[B44] Yan L. Zucker S. Toole B. P. (2005). Roles of the multifunctional glycoprotein, emmprin (basigin; CD147), in tumour progression. *Thromb. Haemost.* 93 199–204. 10.1160/th04-08-0536 15711733

[B45] Yang Y. Lu N. Zhou J. Chen Z. N. Zhu P. (2008). Cyclophilin A up-regulates MMP-9 expression and adhesion of monocytes/macrophages via CD147 signalling pathway in rheumatoid arthritis. *Rheumatology* 47 1299–1310. 10.1093/rheumatology/ken225 18567920 PMC7107241

[B46] Younossi Z. Tacke F. Arrese M. Chander Sharma B. Mostafa I. Bugianesi E. (2019). Global perspectives on nonalcoholic fatty liver disease and nonalcoholic steatohepatitis. *Hepatology* 69 2672–2682. 10.1002/hep.30251 30179269

[B47] Zhang D. W. Zhao Y. X. Wei D. Li Y. L. Zhang Y. Wu J. (2012). HAb18G/CD147 promotes activation of hepatic stellate cells and is a target for antibody therapy of liver fibrosis. *J. Hepatol.* 57 1283–1291. 10.1016/j.jhep.2012.07.042 22878468

